# The effect of sperm concentration on the morphometric traits of spermatozoa of the Arctic fox (*Vulpes lagopus*)

**DOI:** 10.5194/aab-69-227-2026

**Published:** 2026-04-13

**Authors:** Karolina Stasiak, Stanisław Kondracki, Dorota Cygan-Szczegielniak

**Affiliations:** 1 Faculty of Animal Breeding and Biology, Bydgoszcz University of Science and Technology, Mazowiecka 28, 85-084 Bydgoszcz, Poland; 2 Faculty of Agricultural Sciences, University of Siedlce, Prusa 14, 08-110 Siedlce, Poland; 3 Faculty of Technical Sciences, John Paul II University in Biala Podlaska, 95/97 Sidorska St., 21-500 Biała Podlaska, Poland

## Abstract

The ability of spermatozoa to penetrate the egg and their competitiveness in the female reproductive organs may depend on their dimensions and shape, and these are often related to the characteristics of the ejaculate. This study aimed to assess the degree of dependence of the morphological features of spermatozoa on sperm concentration in the ejaculate of the Arctic fox (*Vulpes lagopus*). The research material consisted of ejaculates collected once by manual stimulation from 20 1-year-old foxes. All ejaculates were analysed for standard parameters (volume, sperm concentration, total number of spermatozoa in the ejaculate). Morphometric measurements of spermatozoa were also performed, on the basis of which spermatozoa shape indexes were calculated. It has been shown that the concentration of sperm in the ejaculate affects the morphological characteristics of male Arctic fox sperm. A high concentration of sperm favours the development of minor morphological changes. As the concentration of sperm in the ejaculate increases, the percentage of spermatozoa with major morphological changes decreases. The most common morphological anomaly of sperm in the ejaculates is the “Dag-like” defect, which is most common in ejaculates with the lowest sperm concentration. It has also been shown that, as sperm concentration increases, the dimensions of spermatozoa heads and tails decrease. Sperm concentration affects the shape of Arctic fox sperm. As sperm concentration in ejaculate increases, the ellipticity and elongation indicators decrease, while the head length / total spermatozoa length indicator gradually increases.

## 1 Introduction

Due to sperm morphology and, in particular, the shape of the spermatozoa head, animals can be divided into homomorphic and heteromorphic species (Soler et al., 2014). The former, e.g. sheep, goats, mice, rabbits, and red deer, have constant morphological features both within the same individual and between individuals. In contrast, heteromorphic species show a high degree of spermatozoa polymorphism both within the same ejaculate and between different individuals. Hence, different spermatozoa shapes can be observed in the ejaculates of humans, bulls, dogs, and camelids (Yániz et al., 2015). Spermatozoa are characterized by significant morphological and structural diversity, which influences their functioning and ability to fertilize an egg. Sperm morphology allows us not only to assess sperm structure but also to determine the frequency of normal sperm and those with abnormalities in the head, midpiece, or tail. Morphological anomalies indicate abnormalities in the course of spermatogenesis, especially abnormalities in the final differentiation of sperm following biochemical and morphological changes (including the formation of acrosomes, development of flagella, chromatin condensation, or reorganization of the cell nucleus and cytoplasm). The occurrence of morphological anomalies of spermatozoa indicates the unfavourable influence of genetic (Dotché et al., 2021; Saravia et al., 2007; Wysokińska and Kondracki, 2019) or environmental factors (Petrocelli et al., 2015) that may interfere in the course of morphogenesis. The frequency of morphologically changed spermatozoa also depends on the housing conditions (Kunavongkit et al., 2005) and the male's nutrition (Ezea et al., 2024). The morphology, dimensions, and shape of spermatozoa depend on many factors, including the species of the male; its breed, age, and degree of sexual maturity; and environmental influences (Banaszewska et al., 2011; Stasiak et al., 2018; Yániz et al., 2015). Only spermatozoa with normal structure guarantee a connection with the egg cell (initiation of the acrosome reaction and binding with the oolemma) and, thus, the transfer of genetic information during fertilization (Menkveld et al., 2011; Nagy et al., 2018). However, even spermatozoa with normal morphology may differ in shape. According to Banaszewska et al. (2009), the size and shape of the spermatozoa head depend on the size and shape of the cell nucleus and the surrounding acrosome. In turn, according to Chłopik and Wysokińska (2019), the size of the spermatozoa head depends on the degree of condensation (packing in the nucleus) of chromatin, which, in turn, depends on protamination (replacement of large histone proteins with small protamines) (Sringam et al., 2011). Due to the tight packing of chromatin by protamines, any changes in protamines lead to aberrations in the sperm nucleus (Andraszek et al., 2016). Too loosely packed chromatin and/or DNA fragmentation lead to the appearance of nuclear vacuoles, which negatively affect fertility (Leandri et al., 2013).

Therefore, the quality of spermatozoa can be inferred based on the dimension and shape of the heads (Petrocelli et al., 2015). In turn, the ability of spermatozoa to penetrate the egg cell and their competitiveness in the reproductive organs of females may depend not only on the dimensions and shape of the head but also on the length of the tail (Parisi et al., 2014). It has been shown that differences in the dimension of spermatozoa heads can be used in the diagnosis of male fertility (Severa et al., 2010). The dimension and shape of spermatozoa are often related to the characteristics of the ejaculate (Górski et al., 2017; Stasiak et al., 2018).

An important feature of the ejaculate is the sperm concentration. The number of insemination doses that can be prepared from the ejaculate depends on the sperm concentration. However, it cannot be ruled out that sperm concentration also affects the quality of spermatozoa, including the frequency of morphological changes and the size and shape of spermatozoa, and, therefore, also their motility and ability to fertilize. The high frequency of morphological defects in spermatozoa reduces male fertility (Waberski et al., 2006; Wolf, 2009). This study is an attempt to assess the relationship between the morphological features of sperm (frequency of morphological changes and spermatozoa dimension and shape) and sperm concentration in the ejaculate of male Arctic foxes (*Vulpes lagopus*).

## 2 Materials and methods

### 2.1 Animals and ejaculate collections

A total of 20 1-year-old male Arctic foxes (*Vulpes lagopus*) were randomly selected for the study. The foxes used in the experiment were raised on a fur farm where artificial insemination of foxes is practised. These foxes were housed individually, outdoors in roofed cages. The animals received water at libitum and feed suitable for this species twice daily in accordance with the recommendations of the European Convention (1999). The semen was collected at one time (one day in March, in the middle of the season) from the selected males in the period of their increased sexual activity (which, in the case of the Arctic fox, is from mid-February to mid-April). Ejaculates were collected by manual stimulation following the procedure proposed by Kutzler (2005). In total, 20 ejaculate samples were collected. All of the ejaculates used in the study were classified as normal based on motility exceeding 70 %. After collection, the semen was immediately analysed.

### 2.2 Semen assessment

The following physical characteristics were determined in the freshly collected ejaculates: ejaculate volume (measured using a calibrated test tube), sperm concentration (measured using the Bürker chamber), and total number of spermatozoa in the ejaculate. The collected material was grouped depending on sperm concentration in the ejaculate. Samples from Arctic foxes were divided into three groups: (1) ejaculates with sperm concentration lower than average by at least half the standard deviation (<x±1/2 SD); (2) ejaculates with sperm concentration in the range of ± half of the standard deviation from the mean (x±1/2 SD); and (3) ejaculates with sperm concentration higher than the mean by at least half the standard deviation (>x±1/2 SD).

### 2.3 Sperm morphology and morphometry

Microscopic preparations made from ejaculate samples were stained using eosin and gentian violet (Kondracki et al., 2017). Microscopic examination of the preparations was carried out using immersion objectives at 100 times objective magnification and a light microscope, namely the Nikon Eclipse 50i (Japan). In each specimen, the morphologies of 500 spermatozoa were analysed, and sperm with normal morphology, as well as sperm with major and minor defects, were identified. Additionally, for each slide, morphometric measurements of 15 randomly selected spermatozoa characterized by normal morphology were taken (the cell membrane status was not considered). Altogether, 300 measurements of spermatozoa were taken. Measurements were taken using a system for digital image analysis (Screen Measurement v. 4.1). The following morphometric parameters of spermatozoa were analysed: head length – L, head width – W, perimeter of the head (length of the boundary limiting the spermatozoa head) – P, head area (measuring the area limited by a curve extending along the perimeter) – A, tail length – T, and total spermatozoa cell length – C. Data from morphometric measurements were used to calculate indices defining the shape of the spermatozoa: ellipticity (L / W), elongation [(L-W) / (L + W)], roughness [4π(A / P2)], regularity [π⋅(L ⋅ W / 4 ⋅ A)], ratio of spermatozoa head width to length (W / L), ratio of head length to total spermatozoa length (L / C), ratio of spermatozoa head length to tail length (L / T), ratio of tail length to total spermatozoa length (T / C), ratio of spermatozoa head perimeter to total spermatozoa length (P / C), ratio of spermatozoa head area to total spermatozoa length (A / C), and ratio of spermatozoa head length and width to total spermatozoa length [(L ⋅ W) / C].

### 2.4 Statistical analysis

Most of the data did not meet the requirements of normal distribution, as verified by the Shapiro–Wilk test, or the requirements of homogeneity of variance, as measured with Levene's test, necessary for parametric tests. Therefore, in order to investigate the significance of differences between multiple independent groups, we used non-parametric analysis (non-parametric ANOVA), the Kruskal–Wallis test, and the median test, as well as multiple comparisons of mean ranks for all samples. The obtained data were processed using STATISTICA 13.1 software (StatSoft, Poland).

## 3 Results

The main characteristics of ejaculates collected from selected Arctic foxes are presented in Table 1. The analysed data indicate that ejaculates in group III had a significantly higher concentration and total number of spermatozoa compared to the other groups (p<0.05). The same ejaculates had a smaller volume by 0.36 cm^3^ compared to group I and by 0.62 cm^3^ compared to group II. Ejaculates with the highest sperm concentration (group III) were characterized by the lowest frequency of spermatozoa with major defects. In this group of ejaculates, the average frequency of spermatozoa with major morphological changes was 11.49 % lower compared to group I and 7.79 % lower compared to group II (p<0.05). Spermatozoa with minor changes occurred most often in ejaculates from groups II and III, with medium and high sperm concentration (above $60\times 10^{6}$ cm^−3^). In these ejaculates, the average frequency of spermatozoa with minor changes was 12.46 % and 11.04 %, respectively, and was over 6 % higher than in group I, with the lowest sperm concentration (p<0.05).

**Table 1:** Basic characteristics of ejaculates of Arctic foxes.

Major traits of ejaculates		Group I	Group II	Group III
		$<60\times 10^{6}$	60–$170\times 10^{6}$	$>170\times 10^{6}$
		spermatozoa cm^−3^	spermatozoa cm^−3^	spermatozoa cm^−3^
Number of ejaculates		7	7	6
Ejaculate volume (cm^3^)	$\bar{x}$	0.80^a^	0.91^b^	0.44^c^
	Me	0.85	1.00	0.38
	SD	0.21	0.34	0.24
Sperm concentration ($\times 10^{6}$ cm^−3^)	$\bar{x}$	34.81^a^	94.49^b^	292.77^c^
	Me	30.50	99.75	310.50
	SD	16.47	22.49	87.20
Total number of spermatozoa	$\bar{x}$	286.39^a^	815.79^b^	1191.46^c^
	Me	238.34	727.50	1164.38
	SD	169.14	298.76	476.66
Percentage of normal spermatozoa (%)	$\bar{x}$	77.58^a^	73.71^b^	82.92^a^
	Me	85.50	85.80	88.20
	SD	21.58	19.97	11.26
Percentage of spermatozoa with major changes (%)	$\bar{x}$	17.53^a^	13.83^b^	6.04^c^
	Me	6.60	8.40	6.40
	SD	20.94	10.52	1.15
Percentage of spermatozoa with minor changes (%)	$\bar{x}$	4.90^a^	12.46^b^	11.04^b^
	Me	5.70	7.60	5.60
	SD	2.47	13.46	10.52

Values marked in the same row with different letters (a, b, c) are statistically significantly different at p<0.05, $\bar{x}$ denotes the mean, Me denotes the median, and SD denotes the standard deviation.

The frequency of individual morphological changes in ejaculates differing in terms of sperm concentration is presented in Table 2.

**Table 2:** Frequency of the morphological defects of spermatozoa in Arctic fox ejaculates (%).

Morphological spermatozoa defects			Group I	Group II	Group III
			$<60\times 10^{6}$	60–$170\times 10^{6}$	$>170\times 10^{6}$
			spermatozoa cm^−3^	spermatozoa cm^−3^	spermatozoa cm^−3^
Number of ejaculates			7	7	6
Major sperm defects	Underdeveloped	$\bar{x}$	0.48^a^	0.34^b^	0.16^c^
		Me	0.5	0.4	0.0
		SD	0.41	0.35	0.20
	Double forms	$\bar{x}$	0.30^a^	0.34^a^	0.12^b^
		Me	0.2	0.4	0.0
		SD	0.32	0.28	0.16
	Lost acrosome	$\bar{x}$	0.67^a^	0.43^b^	0.44^b^
		Me	0.5	0.4	0.0
		SD	0.70	0.39	0.55
	Proximal droplet	$\bar{x}$	0.15^a^	0.14^a^	0.20^a^
		Me	0.0	0.0	0.0
		SD	0.40	0.17	0.40
	Distal cytoplasmic	$\bar{x}$	1.13^a,b^	1.09^a^	1.48^b^
	droplet	Me	0.9	0.8	1.4
		SD	1.18	0.93	1.01
	Strongly coiled	$\bar{x}$	14.80^a^	11.40^a^	3.36^b^
	or folded tail	Me	4.1	5.0	4.0
	(Dag-like defect)	SD	20.51	11.24	1.83
Minor spermatozoa defects	Free normal heads	$\bar{x}$	0.15^a^	0.57^b^	0.12^a^
		Me	0.0	0.0	0.0
		SD	0.22	1.02	0.24
	Simple bent tail	$\bar{x}$	3.85^a^	9.63^b^	9.00^b^
		Me	5.2	5.0	4.0
		SD	2.26	12.75	10.62
	Terminally coiled tail	$\bar{x}$	0.80^a^	2.20^b^	0.96^a^
		Me	0.5	1.0	1.0
		SD	0.67	1.51	0.60

Values marked in the same row with different letters (a, b, c) are statistically significantly different at p<0.05, $\bar{x}$ denotes the mean, Me denotes the median, and SD denotes the standard deviation.

**Figure 1:**
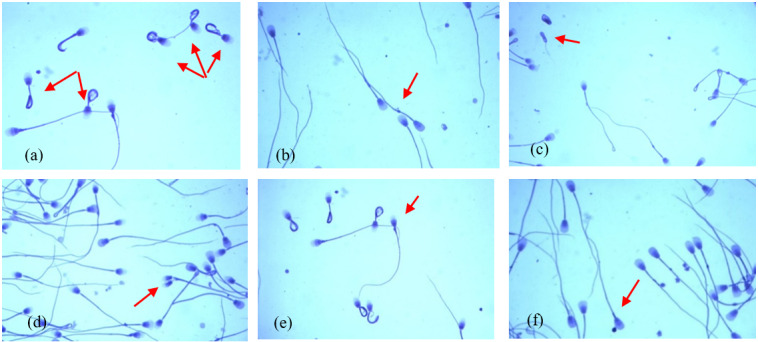
The most common major morphological defects of spermatozoa in the semen of Arctic foxes: **(a)** strongly coiled or folded tail (Dag-like defect), **(b)** distal cytoplasmic droplet, **(c)** underdeveloped, **(d)** double forms, **(e)** lost acrosome, **(f)** proximal droplet.

These data show that the most common morphological spermatozoa anomaly in the ejaculates of male Arctic foxes is the “Dag-like” defect (Fig. 1a). Spermatozoa with a Dag-like defect were most often found in ejaculates with the lowest sperm concentration (group I). In these ejaculates, the frequency of sperm with a Dag-like defect was 14.80 % and was 3.36 % higher than in group II and 11.44 % higher than in group III. Intergroup differences were significant at p<0.05. Among the main morphological changes, spermatozoa with a distal cytoplasmic droplet were also relatively common (Fig. 1b). However, the frequency of spermatozoa with a distal cytoplasmic droplet was much lower than the frequency of spermatozoa with a Dag-like defect and ranged from 1.09 % in the ejaculates from group II to 1.48 % in the ejaculates from group III (p<0.05). In the analysed Arctic fox ejaculates, small amounts of spermatozoa with other major defects were also found, such as underdeveloped (Fig. 1c), double forms (Fig. 1d), lost acrosome (Fig. 1e), and proximal droplet (Fig. 1f). The frequency of spermatozoa showing these major changes was much lower and generally did not exceed 0.70 %.

**Figure 2:**
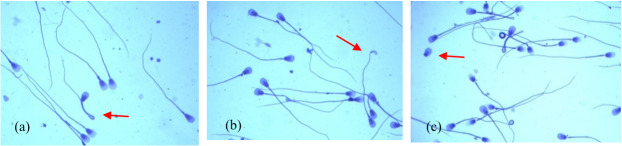
The most common minor morphological defects of spermatozoa in the semen of Arctic foxes: **(a)** simple bent tail, **(b)** terminally coiled tail, **(c)** free normal heads.

The analysed ejaculates also contained spermatozoa showing minor morphological anomalies. The dominant form of subordinate lesions was sperm with a simple bent tail (Fig. 2a). Spermatozoa with a terminally coiled tail (Fig. 2b) and with free normal heads (Fig. 2c) were also relatively common. The largest number of spermatozoa with these changes was found in group-II ejaculates, in which the sperm concentration ranged from 60 to $170\times 10^{6}$ cm^−3^ (p<0.05).

The morphometric parameters of Arctic fox spermatozoa are presented in Table 3.

**Table 3:** Morphometric parameters of spermatozoa from Arctic foxes depending on sperm concentration in the ejaculate.

Variable		Group I	Group II	Group III
		$<60\times 10^{6}$	60–$170\times 10^{6}$	$>170\times 10^{6}$
		spermatozoa cm^−3^	spermatozoa cm^−3^	spermatozoa cm^−3^
Number of ejaculates		7	7	6
Tail length (µm)	$\bar{x}$	66.68^a^	66.36^a,b^	65.03^b^
	Me	67.15	65.89	64.81
	SD	4.05	3.87	3.37
Spermatozoa head length (µm)	$\bar{x}$	7.40^a^	6.80^b^	6.50^c^
	Me	7.41	6.81	6.50
	SD	0.66	0.55	0.39
Spermatozoa head width (µm)	$\bar{x}$	4.53^a^	4.27^b^	4.24^b^
	Me	4.53	4.23	4.25
	SD	0.37	0.29	0.26
Total spermatozoa length (µm)	$\bar{x}$	73.34^a^	72.81^b^	71.24^b^
	Me	74.71	72.63	71.45
	SD	4.11	3.96	3.42
Head perimeter (µm)	$\bar{x}$	20.51^a^	19.04^b^	18.39^c^
	Me	20.42	18.93	18.33
	SD	1.60	1.24	0.81
Head area (µm^2^)	$\bar{x}$	27.93^a^	24.25^b^	23.00^c^
	Me	27.49	23.96	22.72
	SD	4.31	2.96	2.02

Values marked in the same row with different letters (a, b, c) are statistically significantly different at p<0.05, $\bar{x}$ denotes the mean, Me denotes the median, and SD denotes the standard deviation.

The statistical analysis performed showed significant differences between the groups in all spermatozoa dimensions included in the study (p≤0.05). In ejaculates with the lowest sperm concentration (group I), spermatozoa clearly had the largest heads and the longest tails. As sperm concentration in the ejaculate increased, spermatozoa dimensions decreased significantly (p<0.05). The total spermatozoa length was also the highest in the ejaculates of group I. This amounted, on average, to 73.34 µm and was 0.53 and 2.10 µm longer, respectively, than the total spermatozoa length from the ejaculates of groups II and III (p<0.05). The data (Table 4) show that the sperm concentration in the ejaculate has a significant impact on the shape of Arctic fox spermatozoa. As sperm concentration in ejaculate increased, the indicators of ellipticity and elongation decreased significantly, while the head length / total spermatozoa length indicator gradually increased. In all cases, the differences between groups were large and were statistically confirmed (p<0.05). This clearly shows that an increase in sperm concentration in ejaculate results in a change in the shape of spermatozoa heads from more rounded to more elongated. The regularity and roughness indicators changed to a much lesser extent. However, in ejaculates with the highest sperm concentration (group III), spermatozoa heads were characterized by less regularity and, at the same time, greater roughness than spermatozoa heads from the ejaculates from group I, with the lowest sperm concentration (p<0.05). Table 4 also shows that, as sperm concentration in ejaculate increases, the following indicators decrease: head length / tail length; head length / total spermatozoa length; spermatozoa head perimeter / total spermatozoa length; spermatozoa head area / total spermatozoa length; and, in particular, the spermatozoa head length and width / total spermatozoa length indicator (p<0.05).

**Table 4:** Morphometric indexes of spermatozoa from Arctic foxes depending on sperm concentration in the ejaculate.

Variable		Group I	Group II	Group III
		$<60\times 10^{6}$	60–$170\times 10^{6}$	$>170\times 10^{6}$
		spermatozoa cm^−3^	spermatozoa cm^−3^	spermatozoa cm^−3^
Number of ejaculates		7	7	6
Ellipticity	$\bar{x}$	1.63^a^	1.59^b^	1.54^c^
	Me	1.63	1.57	1.52
	SD	0.12	0.13	0.12
Elongation	$\bar{x}$	0.24^a^	0.23^b^	0.21^c^
	Me	0.24	0.22	0.21
	SD	0.03	0.04	0.04
Regularity	$\bar{x}$	0.95^a^	0.94^b^	0.94^b^
	Me	0.95	0.94	0.94
	SD	0.01	0.01	0.01
Roughness	$\bar{x}$	0.83^a^	0.84^a^	0.85^b^
	Me	0.83	0.84	0.85
	SD	0.03	0.03	0.03
Head width / head length (%)	$\bar{x}$	61.67^a^	62.75^a^	65.39^b^
	Me	61.34	63.13	65.61
	SD	4.11	5.06	5.12
Head length / total	$\bar{x}$	10.07^a^	9.43^b^	9.11^c^
spermatozoa length (%)	Me	10.21	9.38	9.11
	SD	0.91	0.87	0.68
Head length / tail length (%)	$\bar{x}$	11.21^a^	10.43^b^	10.03^c^
	Me	11.37	10.35	10.03
	SD	1.13	1.06	0.82
Tail length / total	$\bar{x}$	89.93^a^	90.56^b^	90.89^c^
spermatozoa length (%)	Me	89.79	90.62	90.89
	SD	0.91	0.87	0.68
Spermatozoa head perimeter /	$\bar{x}$	27.87^a^	26.38^b^	25.76^b^
total spermatozoa length (%)	Me	27.99	26.11	25.67
	SD	2.27	2.08	1.58
Spermatozoa head area / total spermatozoa length (%)	$\bar{x}$	38.49^ *a*^	33.73^b^	32.21^b^
	Me	37.93	33.65	31.79
	SD	5.47	4.18	3.07
Product of spermatozoa head	$\bar{x}$	46.51^a^	40.51^b^	38.61^b^
length and width / total	Me	45.99	40.42	38.11
spermatozoa length (%)	SD	6.83	5.18	3.77

Values marked in the same row with different letters (a, b, c) are statistically significantly different at p<0.05, $\bar{x}$ denotes the mean, Me denotes the median, and SD denotes the standard deviation.

## 4 Discussion

With increasing sperm concentration, the total number of spermatozoa increases dynamically. The increase in the total number of spermatozoa in ejaculates with high sperm concentration was very large and indicates a close relationship between the sperm concentration in the ejaculate and the number of spermatozoa excreted (Table 1). This relationship was confirmed in the work of Kozdrowski and Dubiel (2004), who examined the semen of wild boars (*Sus scrofa* L.). It was then found that both parameters of semen quality are directly proportional to each other (r = 0.77). However, it is worth paying attention to the fact that, with the increase in sperm concentration, the volume of ejaculates decreased. It seems that the volume of the ejaculate is inversely proportional to the sperm concentration in the ejaculate. Similar relationships have also been shown in other studies Smital, 2009; Kondracki et al., 2020). Ejaculates from group I, with the lowest sperm concentration, were characterized by the highest percentage of spermatozoa with major morphological changes (17.53 %). As the sperm concentration in the ejaculate increased, the percentage of spermatozoa with major morphological changes decreased. This was mainly due to the presence of spermatozoa with aberrations of the tails resembling the so-called Dag or Dag-like defect (Table 2). To be certain that the strongly coiled tails (Fig. 1a) (causing a significant reduction in male fertility) are characterized by the Dag defect, ultrastructural studies (scanning electron microscopy (SEM) and transmission electron microscopy (TEM)) would be necessary as they are the only methods that reveal abnormalities in the axonemal structures and lesions in the mitochondrial sheath (Parkinson, 2004; Molnar et al. 2001). In this research, the frequency of spermatozoa with a Dag-like defect decreased dynamically with the increase in sperm concentration in the ejaculate. The Dag defect is also often found in boar semen. Some studies show that, in the semen of boars, the frequency of spermatozoa with the Dag defect also decreases with the increase in sperm concentration in the ejaculate (Kondracki et al., 2020). However, the frequency of spermatozoa with a proximal droplet, considered to be the most important main morphological defect (Waberski et al., 2006), and the frequency of spermatozoa with a distal cytoplasmic droplet (indicating disorders during the maturation of sperm in the epididymis) (Parkinson, 2004) did not decrease with increasing sperm concentration in the ejaculate and even increased slightly (Table 2). A similar effect of sperm concentration was found in studies on boar semen (Kondracki et al., 2020). Therefore, it seems that an increasing sperm concentration reduces the occurrence of the Dag-like defect but favours the occurrence of spermatozoa with a proximal droplet and with a distal cytoplasmic droplet.

Morphological features influence the ability of spermatozoa to undergo acrosome reaction, which is reflected in male fertility (Menkveld et al., 2003). Only spermatozoa with normal morphology have a chance to reach the egg cell and initiate oocyte activation (Menkveld et al., 2011). Major spermatozoa malformations are most often the result of disorders in the course of spermatogenesis. They usually occur long before the expulsion of the ejaculate and are often associated with damage to the chromatin structure (Enciso et al., 2011). Abnormalities in the chromatin structure most often occur during the process of spermiogenesis (Andrabi, 2007). Fischer et al. (2003) showed a positive correlation between DNA damage and the frequency of spermatozoa with a protoplasmic droplet and male fertility, and, in subsequent studies, López-Fernandez et al. (2008) confirmed this in boar semen. Disturbances in the course of spermatogenesis result in a reduction in the number of spermatozoa produced and favour the production of spermatozoa with major morphological changes. This study documented the relationship between the frequency of major morphological changes and the sperm concentration in the ejaculates of Arctic foxes. It is possible that the low sperm concentration and, at the same time, the increased frequency of major morphological changes are the result of the same processes that disrupt the course of spermatogenesis.

The percentage of spermatozoa with minor changes is completely different. The frequency of spermatozoa with minor morphological changes was clearly higher in ejaculates with a high sperm concentration (groups II and III). Minor morphological changes in spermatozoa occur in the final, pre-ejaculatory phase of spermatogenesis, when sperm are already in the output tracts of the male reproductive system (Briz et al., 1996). A high sperm concentration in the ejaculate is also associated with a high concentration of sperm in the epididymis just before ejaculation. When there is a high spermatozoa density in the epididymis, conditions favour the development of minor morphological changes in spermatozoa.

The results of this study clearly document the dependence of spermatozoa dimensions on sperm concentration in the ejaculates of male Arctic foxes. The largest dimensions were found in spermatozoa from ejaculates with the lowest sperm concentration (group I). As sperm concentration in the ejaculate increased, spermatozoa dimensions decreased (Table 3). The dependence of spermatozoa dimensions on their concentration in the ejaculate was also found in the studies of ejaculates from male domestic pigs (Kondracki et al., 2020). As the sperm concentration in the ejaculates of male Arctic foxes increased, the size of the spermatozoa head (in terms of both length and width, as well as perimeter and area), tail length, and total spermatozoa length decreased. In turn, Rijsselaere et al. (2004) found that, in the ejaculates from dogs with a lower sperm concentration, spermatozoa had shorter and narrower heads (with a smaller perimeter and area) than spermatozoa in the ejaculates with a higher sperm concentration. Therefore, there are varying data indicating the dependence of sperm dimensions on their concentration in the ejaculates of male mammals. This is perhaps influenced by the breed of the animals studied. Such conclusions result from research conducted on boars (Kondracki et al., 2020), which showed that the dimensions of spermatozoa from Landrace boars (length of the tail, area and perimeter of the spermatozoa head) are inversely proportional to the sperm concentration in the ejaculate, while, in the case of Large White boars, such a relationship was not confirmed.

The dimensions of spermatozoa are important for their motility, which determines the ability of spermatozoa to fertilize an egg cell, and there are studies indicating the relationship between spermatozoa dimensions and their motility (Iwanina and Kondracki, 2019). The relationship between spermatozoa size and male fertility has been the subject of much research (Casey et al., 1997; Hirai et al., 2001; Katz et al., 1986), and most of these studies have shown that smaller spermatozoa sizes promote male fertility. According to Waheed et al. (2015), spermatozoa with a small head (i.e. small width, perimeter, and area) are typical of fertile stallions. Small and shorter spermatozoa heads also characterize the semen of boars with high fertility (Hirai et al., 2001). Similar observations have also been made in studies conducted on human semen, which showed that spermatozoa of men with high fertility had smaller heads and a smaller ratio of spermatozoa head length to width than spermatozoa of men with reduced fertility (Katz et al., 1986). In the context of these studies, it can be assumed that Arctic fox (*Vulpes lagopus*) spermatozoa with the smallest head size will also have a greater fertilization capacity.

The speed of spermatozoa movement depends not only on the size of the spermatozoa head but also on the length of its tail. Research shows that the length of spermatozoa is positively correlated with the speed of their movement, and so spermatozoa with longer tails are faster and can reach the egg faster (Gomendio and Roldan, 2008; Malo et al., 2006). The length of the spermatozoa insert is important for the energy produced in the mitochondria inside it (Bierła et al., 2007), and this affects the movements of the tail and, thus, the strength and speed of spermatozoa movement (Noorafshan and Karbalay-Doust, 2010). Spermatozoa with longer tails have greater motor skills and, therefore, a greater chance of penetrating the egg (Gomendio and Roldan, 2008).

The spermatozoa sizes of male Arctic foxes found in this study turned out to be similar to those obtained in the studies of Andraszek et al. (2020), which showed that the average length of the spermatozoa head of male Arctic foxes is 6.72 µm, and the width of the head is 4.54 µm, while, in our own research, the length of the spermatozoa head was in the range of 6.50–7.40 µm, and its width was in the range of 4.24–4.53 µm. These dimensions, however, differ significantly from the dimensions of male domestic dog spermatozoa found in the studies of Rijsselaere et al. (2007), where the dog spermatozoa heads were significantly smaller. The length of dog spermatozoa heads was smaller by approximately 0.6–0.81 µm, and the width was smaller by approximately 0.37–0.65 µm compared to the spermatozoa heads of male Arctic foxes from the present study. Male Arctic fox spermatozoa tails were approximately 15.88–18.22 µm shorter compared to the dog spermatozoa. It seems that information about spermatozoa size can be treated as a specific biomarker for identifying the species or breed of a male (Stasiak et al., 2021). Information about Arctic fox (*Vulpes lagopus*) semen can therefore be used in the breeding of red foxes (*Vulpes vulpes*), as well as in the breeding of related species (Soler et al., 2017).

The results of this study show that the concentration of sperm in the ejaculates of male Arctic foxes also affects the shape of the spermatozoa. It has been shown that, as sperm concentration in ejaculate increases, the ellipticity and elongation indicators decrease, while the head length / total spermatozoa length indicator increases. The ratio of the length of the spermatozoa head to the length of the tail is recommended to assess the relationship between sperm morphology and their motility (Humphries et al., 2008), which has been empirically confirmed (Simpson et al., 2014). The results of this study also show that, as sperm concentration in ejaculate increases, the following indicators decrease: head length / tail length; head length / total spermatozoa length; spermatozoa head perimeter / total spermatozoa length; spermatozoa head area / total spermatozoa length; and, in particular, the product of spermatozoa head length and width / total spermatozoa length indicator (Table 4). The shape of the head may influence the hydrodynamics of spermatozoa movement (Gomendio and Roldan, 2008; Thurston et al., 2001). According to Gage (1998), spermatozoa with greater movement efficiency have more slender and oval heads. The data of Malo et al. (2006) show that spermatozoa with elongated heads are faster than spermatozoa with rounded heads. The ratio of the length of the spermatozoa head to the width of the head, which describes the elongation of the head, is correlated with the speed of spermatozoa movement, which has been confirmed in red deer (Malo et al., 2006).

The shape of spermatozoa heads is indicated by the ellipticity index, expressed as the ratio of the length to the width of the spermatozoa head. The higher the ellipticity value, the thinner and more elongated the spermatozoa head. The data from this study clearly show that, as sperm concentration in ejaculate increases, the ellipticity value decreases. This means that spermatozoa from ejaculates with low sperm concentration have thinner and longer heads, which, according to Gil et al. (2009), may indicate a greater capacity for progressive movement. It is worth noting that the results obtained in these studies indicate that spermatozoa from male Arctic foxes have a relatively thin and elongated head. The spermatozoa heads of male Arctic foxes from our own studies were narrower and less rounded than those presented in the work of Andraszek et al. (2020). The research by Noorafshan and Karbalay-Doust (2010) shows that the shape of the spermatozoa tail affects male fertility. The spermatozoa tails of fertile males are straight, while, in males with reduced fertility, they are bent at the place where the cytoplasmic droplet is retained. The presence of this defect in semen indicates a disturbed process of spermatozoa maturation, which occurs during the movement of spermatozoa through successive sections of the epididymis (Brodzki et al., 2015). A maturation disorder in the initial stage of spermatogenesis causes the appearance of a protoplasmic droplet in a proximal position (which is one of the main defects of the spermatozoa), while, in the final stage of spermatogenesis, it leads to the formation of a protoplasmic droplet in a distal position (the presence of which is treated as a minor defect). Even a small percentage of spermatozoa with a protoplasmic drop in a proximal position can reduce sperm motility and male fertility.

## 5 Conclusions

1. As the sperm concentration in the ejaculate increases, the total number of spermatozoa increases dynamically. The volume of the ejaculate, however, remains inversely proportional to the sperm concentration in the ejaculate.
2. A high sperm concentration in the ejaculate promotes the development of subordinate morphological changes. As the sperm concentration in the ejaculate increases, the percentage of spermatozoa with major morphological changes decreases.
3. The most common morphological anomaly of spermatozoa in Arctic fox ejaculates is the Dag-like defect, which occurs most frequently in ejaculates with the lowest sperm concentration.
4. The dimensions of spermatozoa in male Arctic foxes depend on the sperm concentration in the ejaculate. As the sperm concentration in the ejaculate increases, the dimension of the spermatozoa heads and tails decreases.
5. Sperm concentration has an impact on the shape of Arctic fox spermatozoa. As sperm concentration in ejaculate increases, the ellipticity and elongation indices decrease, while the head length / total spermatozoa length index gradually increases.

## Data Availability

The datasets used and/or analysed during the current study are available from the corresponding author on reasonable request.
